# QT interval and repolarization dispersion changes during the administration of hydroxychloroquine/chloroquine with/without azithromycin in early COVID 19 pandemic: A prospective observational study from two academic hospitals in Indonesia

**DOI:** 10.1002/joa3.12623

**Published:** 2021-08-28

**Authors:** Rizki A. Gumilang, Siswanto, Vita Y. Anggraeni, Ika Trisnawati, Eko Budiono, Anggoro B. Hartopo

**Affiliations:** ^1^ Department of Cardiology and Vascular Medicine Faculty of Medicine Public Health and Nursing Universitas Gadjah Mada Universitas Gadjah Mada Academic Hospital Yogyakarta Indonesia; ^2^ Department of Physiology Faculty of Medicine Public Health and Nursing Universitas Gadjah Mada Universitas Gadjah Mada Academic Hospital Yogyakarta Indonesia; ^3^ Division of Cardiology Department of Internal Medicine Faculty of Medicine Public Health and Nursing Universitas Gadjah Mada Dr. Sardjito Hospital Yogyakarta Indonesia; ^4^ Division of Pulmonology Department of Internal Medicine Faculty of Medicine Public Health and Nursing Universitas Gadjah Mada Dr. Sardjito Hospital Yogyakarta Indonesia; ^5^ Department of Cardiology and Vascular Medicine Faculty of Medicine Public Health and Nursing Universitas Gadjah Mada Dr. Sardjito Hospital Yogyakarta Indonesia

**Keywords:** arrhythmia, azithromycin, chloroquine, COVID‐19, electrocardiography, hydroxychloroquine

## Abstract

**Background:**

Hydroxychloroquine/chloroquine (HCQ/CQ) treatment for COVID‐19 was associated with QT interval prolongation and arrhythmia risks. This study aimed to investigate QTc interval and ventricular repolarization dispersion changes, as markers of arrhythmia risks, after HCQ/CQ administration with/without azithromycin (AZT) during COVID‐19 pandemic.

**Methods:**

A prospective observational study was performed in two academic hospitals in Indonesia. Adult patients who received HCQ/CQ alone and HCQ/CQ + AZT concomitant treatments for COVID‐19 infection were enrolled. Baseline and post HCQ/CQ treatment electrocardiograms were obtained. Baseline and post HCQ/CQ treatment QT interval by Bazett (B‐QTc) and Fridericia (F‐QTc) formulas and ventricular repolarization dispersion indices by Tpeak‐Tend (Tp‐e) interval and Tpeak‐Tend/QT (Tp‐e/QT) ratio were calculated and analyzed.

**Results:**

The study enrolled 55 (HCQ/CQ alone) and 77 subjects (HCQ/CQ + AZT concomitant). F‐QTc interval significantly lengthened in subjects with HCQ/CQ + AZT (mean difference 11.89 ms [*P* = .028]). The incidences of severe B‐QTc and F‐QTc lengthening were 13.1% and 12.3%, B‐QTc and F‐QTc prolongation were 25.4% and 12.3%, and severe B‐QTc and F‐QTc prolongation were 6.2% and 3.2%. Tp‐e interval lengthened significantly from baseline to posttreatment in HCQ/CQ alone and HCQ/CQ + AZT (mean difference 10.83 ms [*P* = .006] and 18.73 ms [*P* < .001], respectively). Tp‐e/QT ratio increased significantly from baseline to posttreatment in HCQ/CQ + AZT concomitant (mean difference 0.035 [*P* < .001]). No fatal arrhytmia occurred.

**Conclusions:**

During COVID‐19 pandemic, HCQ/CQ + AZT concomitant treatment caused significant F‐QTc lengthening, significantly increased Tp‐e interval and increased Tp‐e/QT ratio. HCQ/CQ alone only caused significant increase of Tp‐e interval. Incidences of severe QTc lengthening and prolongation were low in both HCQ/CQ alone and HCQ/CQ + AZT concomitant.

## Graphical abstract

HCQ/CQ + AZT concomitant treatment caused significant F‐QTc lengthening, significantly increased Tp‐e interval and increased Tp‐e/QT ratio. Whereas, HCQ/CQ alone only caused significantly increased Tp‐e interval. The incidences of severe QTc lengthening and severe QTc prolongation, as well as arrhythmia, were similarly low in HCQ/CQ alone and HCQ/CQ + AZT concomitant.

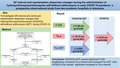

## 1 INTRODUCTION

A novel Severe Acute Respiratory Syndrome Coronavirus‐2 (SARS‐CoV‐2) reached pandemic status in early 2020 due to its fast spreading pneumonia becoming transmitted worldwide.^1^, ^2^ This new disease was acronymized as COVID‐19 (Coronavirus Disease of 2019).^2^ To date, COVID‐19 has afflicted as many as 80 million people and caused more than 1.7 million death globally.^3^ Indonesia reported its earliest cases of COVID‐19 on March 1, 2020 and subsequently the number of cases has increased progressively.^4^, ^5^

At the beginning of this pandemic, several drugs were proposed for the management of COVID‐19 including hydroxychloroquine (HCQ), chloroquine (CQ), and azithromycin (AZT).^6^ Either HCQ or CQ hinders viral replication and reduces viral load, which is reinforced by adding AZT in the treatment regimen.^7^, ^8^ Over time, the evidence shows unconvincing data regarding the effectiveness of this treatment regimen for COVID‐19.^9^, ^10^ The regimens of HCQ/CQ with/without AZT have very limited documented clinical benefit for COVID‐19.^11^, ^12^, ^13^ Therefore, World Health Organization (WHO) recommends against the use of HCQ/CQ in the latest guideline for management of COVID‐19.^14^

Moreover, HCQ and CQ as well as AZT are QT‐prolonging drugs which pose a risk of malignant ventricular arrhythmia, Torsade de Pointes (TdP), and cardiac arrest.^15^, ^16^, ^17^ The synthesis of several studies indicate the increased incidence of corrected QT (QTc) prolongation and TdP in COVID‐19 patients using HCQ/CQ alone or in combination with AZT.^18^, ^19^ Furthermore, in about 13% COVID‐19 patients suffer from QTc prolongation due to this illness.^20^ Similar to what had occurred in other parts of the world, in Indonesia the use of HCQ/CQ in combination with AZT as a treatment regimen for COVID‐19 had been implemented in the national protocol since March 2020.^21^ Despite many evidences of QTc prolongation due to HCQ/CQ administration, there are still insufficient data of other markers of ventricular arrhythmia in relation to this regimen. A Tpeak ‐Tend (Tp‐e) interval and Tpeak‐Tend/QT (Tp‐e/QT) ratio reflect ventricular repolarization dispersion, which are regarded as markers of ventricular arrhythmia and sudden cardiac death.^22^, ^23^ These parameters, in addition to QTc changes, may indicate the cardiac toxicities of HCQ/CQ in COVID‐19 treatment regimen. Despite warnings regarding cardiac side effects, evidence at that time was scarce and our physicians were accustomed to administering HCQ/CQ for malaria without any significant side effects. Our data on QTc interval and ventricular repolarization dispersion changes due to HCQ/CQ treatment for COVID‐19 will provide additional data from Southeast Asian countries, which are still underrepresented in scientific literatures, despite the huge number of HCQ/CQ usage in the region. We conducted a prospective observational study to investigate the changes of QTc interval, Tp‐e interval and Tp‐e/QT ratio, and the incidence of QTc prolongation and cardiac arrhythmias in Indonesian patients who received HCQ/CQ alone or HCQ/CQ + AZT concomitant treatment during COVID‐19 pandemic.

## 2 METHODS

### 2.1 Study design and subjects

This study design was a prospective observational study. Subjects were adult patients enrolled from March 2020 to September 2020. The subjects were patients with the diagnosis of high‐probability‐COVID‐19 according to the national protocol and COVID‐19‐PCR positive hospitalized in hospital wards and intensive care units (ICU) specified for COVID‐19 patients in two academic hospitals affiliated with Universitas Gadjah Mada, namely Dr Sardjito Hospital and Universitas Gadjah Mada Academic Hospital, Yogyakarta, Indonesia.

The subjects were patients receiving HCQ/CQ as one of the treatment regimens for COVID‐19. The decision to treat with HCQ/CQ was based on the clinical decision of the attending physicians and each hospital clinical practice guideline at the time of this study. The inclusion criteria were: (i) patients age ≥18 years old, (ii) patients with high‐probability COVID‐19 or COVID‐19‐PCR positive, (iii) patients hospitalized in hospital wards or ICU, (iv) patients who received HCQ/CQ treatments, and (v) patients agreed, by signing an informed consent form, to participate in this study. The exclusion criteria were: (i) the electrocardiogram could not be obtained in one of the time points, (ii) the HCQ/CQ was prematurely stopped due to non‐cardiac reasons, (iii) pregnant patients, and (iv) the electrocardiogram could not be interpreted due to technical reasons. The study protocol was approved by Medical and Health Research Ethics Committee of the Faculty of Medicine, Public Health and Nursing Universitas Gadjah Mada—Dr Sardjito Hospital and Ethics Committee of Universitas Gadjah Mada Academic Hospital, Yogyakarta, Indonesia.

### 2.2 Subject enrollment and baseline measurements

The diagnosis and treatment protocol for patients with suspected COVID‐19 in our hospitals were based on the national protocol released per March 2020.^21^ At the beginning of the COVID 19 pandemic due to inadequate SARS‐Cov‐2 PCR facilities and the delayed result of PCR, all the patients who fulfilled the high probability COVID‐19 criteria and had symptoms at admission were treated as COVID‐19 patients until otherwise proven by PCR result.^21^ Our hospitals adopted this protocol and implemented it as hospital clinical practice guidance. Our hospitals developed a screening protocol for patients with signs and symptoms suspicious of COVID‐19. Patients with screening results who showed high probability of COVID‐19 were treated in the hospital ward or ICU and were examined by nasopharyngeal swabs for SARS‐CoV‐2 RT‐PCR twice for diagnostics (usually in day 1 and day 2). Confirmed‐COVID‐19‐PCR positive patients were patients with positive result of at least one of SARS‐CoV‐2 RT‐PCR. Subjects with negative results of SARS‐CoV‐2 RT‐PCR were diagnosed as high probability‐COVID‐19‐PCR negative patients. Subjects with either diagnosis were enrolled in the study if they fulfilled the inclusion and exclusion criteria. The disease severity was divided into three categories: mild, moderate, and severe.^21^ Mild disease: uncomplicated respiratory tract infection with nonspecific symptoms (fever, malaise, anorexia, myalgia, sore throat, dyspnea, nasal congestion, headache, dyspepsia, and/or diarrhea). Moderate disease: pneumonia without severe symptoms and no need for supplemental oxygen therapy. Severe disease: pneumonia with at least one severe symptoms, ie respiratory rate ≥30 rate/min, severe respiratory distress, or oxygen saturation <93% at room air or PaO2/FiO2 ratio <300.^21^

The demographic data, history of illness, cardiovascular comorbidities, and current medications were collected and recorded in an electronic case report form on admission and during observation. The laboratory data were obtained from hospital central laboratories. The Tisdale score and its risk categories were calculated based on baseline parameters and divided into three categories: low risk (score <7), moderate risk (score 7‐10), and highrisk (score ≥11).^24^ The disease severity was determined based on the national protocol classification.^21^

### 2.3 Electrocardiogram recording

Twelve‐lead electrocardiogram was obtained at baseline and post HCQ/CQ treatment. Baseline electrocardiogram was performed before commencement of HCQ/CQ. Post HCQ/CQ treatment electrocardiogram was performed at the last day after or within 24 hours of last HCQ/CQ doses. During the administration of the drugs, between baseline and post HCQ/CQ treatment electrocardiogram was performed every day or every other day based on baseline Tisdale risk score or by the discretion of attending physician. The electrocardiogram was recorded using standardized electrocardiograph machines, with standard 12‐lead resting electrocardiogram, paper speed of 25 mm/s, the amplitude of 10 mm/V, and a sampling rate of 250 Hz. In several subjects, the electrocardiogram in the post HCQ/CQ treatment was recorded only in limb leads (lead II) due to the constraint to reduce staff exposure with patients. In this study, both baseline and post HCQ/CQ treatment electrocardiograms were mandatory to be included in the analysis.

### 2.4 Measurement and calculation of QTc interval, Tpeak‐Tend interval, and Tpeak‐Tend/QT ratio

The measurement of QT interval was performed by two cardiologists (R.A.G. and A.B.H.) independently and blindly. The coefficient correlation of the ratings of these two cardiologists was >90%. The QT interval was measured visually from the onset of the first deflection of QRS complex from isoelectric line to the end of T wave. The end of the T wave was determined by the tangent point of its steepest return to isoelectric line. The QT measurement was performed mainly in lead II, if the T‐wave could not be determined in lead II then lead I, V5 or V6 were used. In a case of a bundle‐branch block, the J‐T interval was measured and 120 ms was added to obtain the QT interval duration.^25^ Three beats of QT were measured and mean value was used. All measurements were performed manually by standard calipers and aided by computer. The QTc interval was calculated based on the Bazett's formula and Fridericia's formula. The Tp‐e interval was measured in V5 lead,^26^ and the Tp‐e/QT ratio was measured by dividing Tp‐e interval from V5 lead with measured QT interval as described above.

### 2.5 Treatments and observation

Based on the hospital clinical practice guidance, HCQ/CQ could be given in all disease severities. The HCQ doses were: (i) mild cases: 400 mg q.i.d. orally for 5 days, (ii) moderate cases: 400 mg b.i.d. orally at first day followed by 400 mg q.i.d. or 200 mg b.i.d. for 5‐7 days, and (iii) severe cases: 400 mg q.i.d. orally for 7 days. The CQ doses were: (i) mild cases: 500 mg (phosphate) b.i.d. orally for 5 days, (ii) moderate cases: 500 mg (phosphate) b.i.d. orally for 5‐7 days, and (iii) severe cases: 500 mg (phosphate) b.i.d. orally for 3 days followed by 250 mg b.i.d. orally until 10 days. The combination of HCQ/CQ with AZT (500 mg per oral q.i.d for 3‐5 days) was given concomitantly. However, the decision of HCQ/CQ treatment, concomitantly with/without AZT, and the duration of treatment were made at the discretion of the attending physicians. Other medications were also given by the attending physicians and recorded in our electronic case report form. The decisions of attending physicians were not interfered by this study.

### 2.6 Primary and secondary outcomes

The primary outcomes of the study were the changes of QTc interval, Tp‐e interval, and Tp‐e/QT ratio after HCQ/CQ alone and HCQ/CQ + AZT concomitant treatments. These changes were analyzed by measuring QTc interval, Tp‐e interval, and Tp‐e/QT ratio differences from baseline to post HCQ/CQ treatment.

The secondary outcomes were the incidences of severe QTc lengthening, QTc prolongation, and severe QTc prolongation. A QTc lengthening was defined as any increased of QTc interval from baseline to post HCQ/CQ treatment. A severe QTc lengthening was defined as QTc changes ≥60 ms from baseline to post HCQ/CQ treatment. A QTc prolongation was defined as QTc value >450 ms (males) and QTc value >460 ms (females) at post HCQ/CQ treatment. A severe QTc prolongation was defined as QTc value ≥500 ms at post HCQ/CQ treatments. The presence of any arrhythmias was also reported.

### 2.7 Statistics analysis

The continuous data were reported as mean ± standard deviation (mean ± SD). The categorical data were reported as count and percentage (n (%)). The continuous data were tested for normality by the Kolmogorov–Smirnov test or Shapiro–Wilk test in every group allocated for analysis. This normality test determined the parametric or nonparametric analysis for continuous data. The comparison of continuous data between paired groups (baseline vs posttreatment) was analyzed using paired‐T test or Wilcoxon–Signed rank test. The comparison of continuous data between nonpaired groups (HCQ/CQ alone vs HCQ/CQ + AZT concomitant) was performed with Student T‐test or Mann–Whitney test. The comparison between categorical data was analyzed by chi‐squared test or Fischer exact test. A *P* < .05 was considered as statistically significant. Statistical analyses were performed using SPSS version 22.0 (IBM Corp., USA).

## 3 RESULTS

### 3.1 Baseline characteristics

**FIGURE 1:**
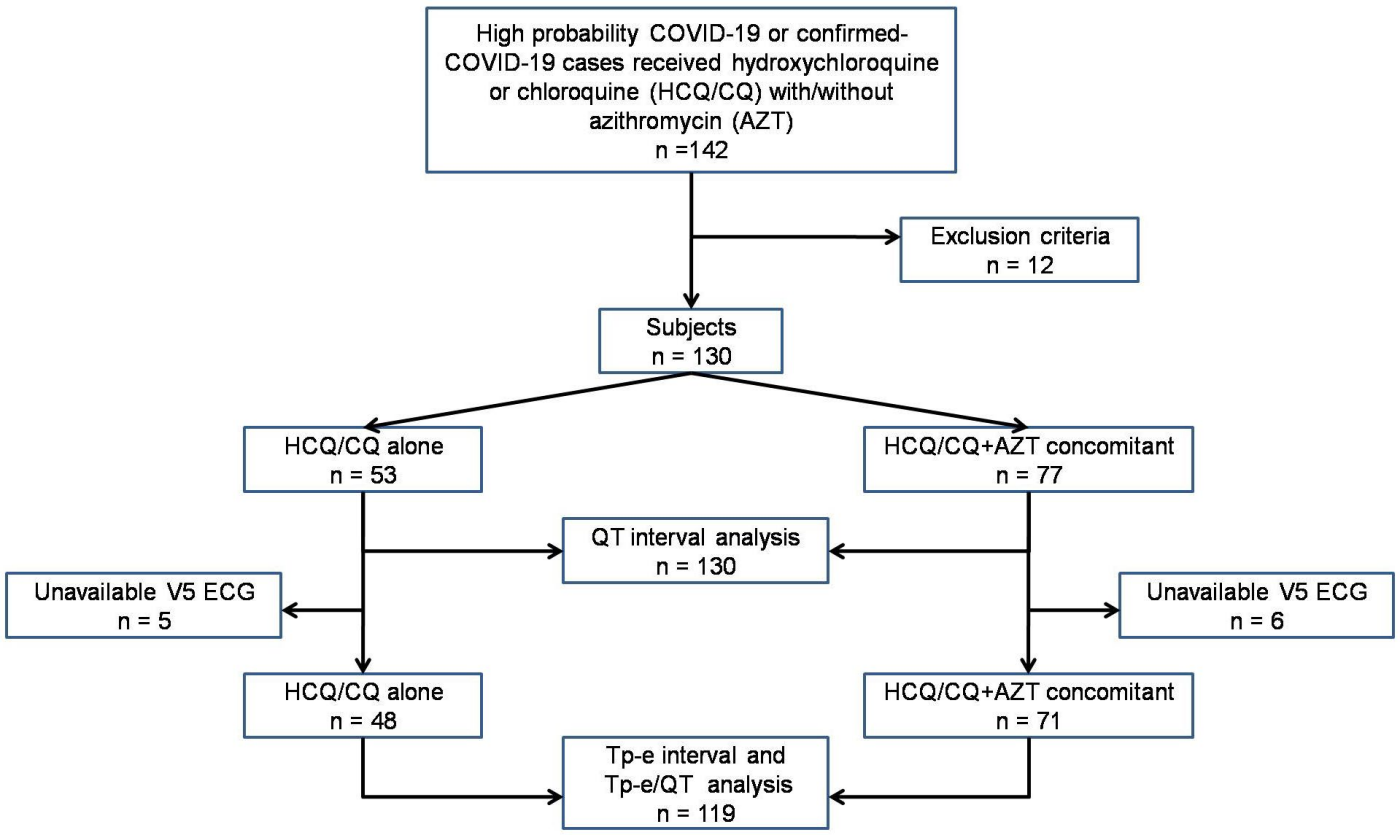
The flowchart of subjects’ enrollment (n = 130) and analysis for QTc interval (n = 130) and for Tp‐e interval and Tp‐e/QT ratio (n = 119)

From 142 patients who satisfied the inclusion criteria, 12 of them were excluded due to incompleteness of electrocardiogram data. Most of the reason of incompleteness was the self‐imposed constraint on exposure or contact with the patients by staffs on duty. In our observational data, all of the 12 excluded patients were clinically well until post HCQ/CQ treatment. Figure 1 shows the flowchart of subjects’ enrollment and analysis.

Table 1 presents the characteristics of the subjects eligible for analysis in this study. Of 130 subjects, mean of age was 48 years old and 60% were males. Subjects with confirmed‐COVID‐19‐PCR positive were 68.5%. The majority of disease severity at diagnosis was moderate disease (40%). The HCQ was more frequently administered than CQ with the mean duration of both treatments was 5.8 ± 1.5 days and cumulative dose was 1816.3 mg. The Tisdale risk category mostly fell into low risk, and only 6.9% were in high‐risk category. The most common cardiovascular comorbidity was hypertension (28.5%).

**TABLE 1:** Baseline characteristics of subjects receiving HCQ/CQ alone and HCQ/CQ + AZT concomitant treatments

Characteristics	Total (n = 130)	HCQ/CQ alone (n = 53)	HCQ/CQ + AZT concomitant (n = 77)	*P* value
Age (years), mean ± SD	48.1 ± 15.8	52.8 ± 15.3	44.9 ± 15.4	.005
Male sex, n (%)	78 (60.0)	34 (64.2)	44 (57.1)	.423
Treatments HCQ/CQ, n (%)				.184^a^
HCQ, n (%)	115 (88.5)	49 (92.5)	66 (85.7)	
CQ, n (%)	15 (11.5)	4 (7.5)	11 (14.3)	
HCQ/CQ duration (days), mean ± SD	5.8 ± 1.5	5.7 ± 1.2	5.8 ± 1.6	.994^b^
HCQ/CQ cum dose (mg), mean ± SD	1816.3 ± 1137.3	1781.1 ± 933.4	1832.5 ± 1259.54	.812^b^
Tisdale score, mean ± SD	6.9 ± 2.1	6.4 ± 2.6	7.2 ± 1.6	.075^b^
Tisdale risk category, n (%)				.762
Low	62 (47.7)	27 (50.9)	35 (45.5)	
Moderate	59 (45.4)	22 (41.5)	37 (48.1)	
High	9 (6.9)	4 (7.5)	5 (6.5)	
COVID 19‐PCR positive, n (%)	89 (68.5)	38 (71.7)	51 (66.2)	.51
Disease severity, n (%)				.016
Mild	50 (38.5)	13 (24.5)	37 (48.1)	
Moderate	52 (40.0)	28 (52.8)	24 (31.2)	
Severe	28 (21.5)	12 (22.6)	16 (20.8)	
Hypertension, n (%)	37 (28.5)	16 (30.2)	21 (27.3)	.717
Diabetes mellitus (%)	24 (18.5)	12 (22.6)	12 (15.6)	.308
Ischemic heart disease, n (%)	4 (3.1)	1 (1.9)	3 (3.9)	.460^a^
Chronic heart failure, n (%)	6 (4.6)	2 (3.8)	4 (5.2)	.528^a^
Chronic kidney disease, n (%)	6 (4.7)	1 (1.9)	5 (6.5)	.215^a^
Lopinavir, n (%)	12 (9.2)	5 (9.4)	7 (9.1)	.947
Favipiravir, n (%)	2 (1.5)	2 (3.8)	0 (0)	.164^a^
Oseltamivir, n (%)	9 (6.9)	3 (5.7)	6 (7.8)	.461^a^
Umifenovir, n (%)	8 (6.2)	5 (9.4)	3 (3.9)	.178^a^
Levofloxacine, n (%)	25 (19.2)	15 (28.3)	10 (13.0)	.029
Moxifloxacine, n (%)	6 (4.6)	4 (7.5)	2 (2.6)	.185
Meropenem, n (%)	17 (13.1)	9 (17.0)	8 (10.4)	.273
Steroids	14 (10.8)	5 (3.8)	9 (6.9)	.243^a^
Dexamethason	7 (5.4)	4 (7.5)	3 (3.9)	
Metilprednisolone	7 (5.4)	1 (1.9)	6 (7.8)	
Heart rate (bpm), mean ± SD	88.75 ± 17.59	86.77 ± 19.79	90.12 ± 15.89	.289
B‐QTc interval	421.32 ± 43.16	416.00 ± 47.21	424.97 ± 40.05	.246
F‐QTc interval	397.13 ± 39.58	393.92 ± 42.62	399.34 ± 37.48	.446
B‐QTc prolongation, n (%)	27 (20.8)	12 (22.6)	15 (19.5)	.662
F‐QTc prolongation, n (%)	8 (6.2)	3 (5.7)	5 (6.5)	.578^a^
B‐QTc severe prolongation, n (%)	6 (4.6)	3 (5.7)	3 (3.9)	.638^a^
F‐QTc severe prolongation, n (%)	0 (0)	0 (0)	0 (0)	NA

Abbreviations: AZT, azithromycin; bpm, beat per minute; B‐QTc, corrected QT based on Bazett'sformula; CQ, chloroquine; Cum, cumulative; F‐QTc, corrected QT based on Fridericia's formula; HCQ, hydroxychloroquine; NA, not applicable; PCR, polymerase chain reaction; SD, standard deviation.

^a^ Fisher's Exact test.

^b^ Mann–Whitney U‐test.

Fifty‐three subjects (40.8%) had HCQ/CQ treatment alone and 77 subjects (59.2%) received HCQ/CQ + AZT concomitant treatment. Subjects with HCQ/CQ alone were significantly older (mean ± SD: 52.8 ± 15.3 vs 44.9 ± 15.4 years old, *P* = .005) and more fell into moderate disease severity than subjects who received HCQ/CQ + AZT concomitant (28 [52.8%] vs 24 [31.2%], *P* = .016). Levofloxacine drug was prescribed more in subjects HCQ/CQ alone than in HCQ/CQ + AZT concomitant (28.3% vs 13.0%, *P* = .029) with concomitant and concurrent deliveries. Other characteristics at baseline were not significantly different between treatment groups (Table 1).

### 3.2 Primary outcome

The QTc interval lengthened for both B‐QTc and F‐QTc in subjects with HCQ/CQ alone and those received HCQ/CQ + AZT concomitant. The significant QTc lengthening was found in F‐QTc of subjects with HCQ/CQ + AZT concomitant group, with F‐QTc change mean difference of 11.89 ms (*P* = .028). There were no significant differences in the value of baseline QTc interval, posttreatment QTc interval, and QTc interval changes in both treatment groups. Table 2 showed the QTc interval and its changes between groups.

**TABLE 2:** The QTc interval changes from baseline to posttreatment of HCQ/CQ alone and HCQ/CQ + AZT concomitant treatment

Treatment group	Baseline B‐QTc (ms, mean ± SD)	Post HCQ/CQ B‐QTc (ms, mean ± SD)	B‐QTc changes (ΔQTc) (ms, mean [95% CI])	*P* value
HCQ/CQ (n = 53)	416.00 ± 47.21	425.91 ± 51.06	9.91 (−5.97 to 25.78)	.117^a^
HCQ/CQ + AZT (n = 77)	424.97 ± 40.05	432.00 ± 44.68	7.02 (−3.91 to 17.96)	.219^a^
*P* value	.246^b^	.447	.758^b^	

Treatment group	Baseline F‐QTc (ms, mean ± SD)	Post HCQ/CQ F‐QTc (ms, mean ± SD)	F‐QTc changes (ΔQTc) (ms, mean [95% CI])	*P* value
HCQ/CQ (n = 53)	393.92 ± 42.62	406.98 ± 47.27	13.06 (−0.46 to 26.57)	.054^a^
HCQ/CQ + AZT (n = 77)	399.34 ± 37.48	411.23 ± 44.41	11.89 (1.27 to 22.53)	.028^a^
*P* value	.446^b^	.491^c^	.892^b^	

Abbreviations: AZT, azithromycin; B‐QTc, corrected QT based on Bazett'sformula; CI, confidence interval; CQ, chloroquine; F‐QTc: corrected QT based on Fridericia's formula; HCQ, hydroxychloroquine; ms, milliseconds; SD, standard deviation.

^a^ Wilcoxon–Signed Rank test (baseline vs post HCQ/CQ).

^b^ Student's *t*‐test (HCQ/CQ alone vs HCQ/CQ + AZTconcomitant).

^c^ Mann–Whitney U‐test (HCQ/CQ alone vs HCQ/CQ + AZTconcomitant).

For Tp‐e and Tp‐e/QT ratio analysis, there were 119 subjects whom Tp‐e interval can be measured in V5 lead. Eleven subjects were dropped out from Tp‐e interval and Tp‐e/QT ratio analysis due to unavailable Tp‐e interval from V5 lead in either baseline or posttreatment (Figure 1). There were significant changes of Tp‐e interval from baseline to posttreatment of both HCQ/CQ alone and HCQ/CQ + AZT concomitant groups and Tp‐e/QT ratio in the HCQ/CQ + AZT concomitant group (Table 3). In HCQ/CQ alone group, the Tp‐e interval change from baseline to posttreatment mean difference was 10.83 ms (*P* = .006). In HCQ/CQ + AZT concomitant group, the Tp‐e interval change mean difference was 18.73 ms (*P* < .001) and Tp‐e/QT ratio change mean difference was 0.035 (*P* < .001), from baseline to posttreatment. There were no differences in baseline value, posttreatment value, and mean changes of Tp‐e interval and Tp‐e/QT ratio between both groups.

**TABLE 3:** The Tpeak‐Tend (Tp‐e) and Tp‐e/QT ratio from baseline to posttreatment of HCQ/CQ alone and HCQ/CQ + AZT concomitant treatments

Treatment group	Baseline Tp‐e (ms, mean ± SD)	Post HCQ/CQ Tp‐e (ms, mean ± SD)	Mean Tp‐e changes (ΔTp‐e) (ms, 95% CI)	*P* value
HCQ/CQ (n = 48)	85.73 ± 17.48	96.67 ± 23.09	10.83 (3.36‐18.31)	.006^a^
HCQ/CQ + AZT (n = 71)	82.82 ± 18.91	101.55 ± 18.49	18.73 (13.16‐24.30)	<.001^a^
*P* value	.580^b^	.177^b^	.097^b^	

Treatment group	Baseline Tp‐e/QT (mean ± SD)	Post HCQ/CQ Tp‐e/QT (mean ± SD)	Mean Tp‐e/QT changes (ΔTp‐e/QT), (mean changes, 95%CI)	*P* value
HCQ/CQ (n = 48)	0.247 ± 0.054	0.260 ± 0.062	0.016 (−0.004 to 0.037)	.089^a^
HCQ/CQ + AZT (n = 71)	0.239 ± 0.060	0.270 ± 0.055	0.035 (0.020 to 0.050)	<.001^a^
*P* value	.516^c^	.366^c^	.142^c^	

Abbreviations: AZT, azythromycin; CI, confidence interval; CQ, chloroquine; HCQ, hydroxychloroquine; ms, milliseconds; SD, standard deviation.

^a^ Wilcoxon–Signed Rank test (baseline vs post HCQ/CQ).

^b^ Mann–Whitney U‐test (HCQ/CQ alone vs HCQ/CQ + AZT concomitant).

^c^ Student's *t*‐test (HCQ/CQ alone vs HCQ/CQ + AZT concomitant).

### 3.3 Secondary outcomes

In all subjects, the incidence of severe B‐QTc and F‐QTc lengthening was 13.1% and 12.3%, B‐QTc and F‐QTc prolongation were 25.4% and 12.3%, and severe B‐QTc and F‐QTc prolongation were 6.2% and 3.8%, respectively. There were no significant differences in their incidences between subjects with HCQ/CQ alone and HCQ/CQ + AZT concomitant groups (Table 4).

**TABLE 4:** Incidences of severe QTc lengthening, QTc prolongation, and severe QTc prolongation, based on Bazett's (B‐QTc) and Fridericia (F‐QTc) formula, between subjects with HCQ/CQ alone and HCQ/CQ + AZT concomitant treatment

QTc categorization	Total (n = 130)	HCQ/CQ alone (n = 53)	HCQ/CQ + AZT concomitant (n = 77)	*P* value
Severe B‐QTc lengthening, n (%)	17 (13.1)	8 (15.1)	9 (11.7)	.571
Severe F‐QTc lengthening, n (%)	16 (12.3)	6 (11.3)	10 (13.0)	.776
B‐QTc prolongation, n (%)	33 (25.4)	16 (30.2)	17 (22.1)	.296
F‐QTc prolongation, n (%)	16 (12.3)	7 (13.2)	9 (11.7)	.796
Severe B‐QTc prolongation, n (%)	8 (6.2)	4 (7.5)	4 (5.2)	.422^a^
Severe F‐QTc prolongation, n (%)	5 (3.8)	3 (5.7)	2 (2.6)	.328^a^

Abbreviations: AZT, Azythromycin; B‐QTc, corrected QT based on Bazett's formula; CQ, chloroquine; F‐QTc, corrected QT based on Fridericia's formula; HCQ, hydroxychloroquine.

^a^ Fisher's Exact Test.

The characteristics associated with severe B‐QTc and F‐QTc lengthening were depicted in Table 5. Severe B‐QTc lengthening had lower baseline B‐QTc interval, longer post B‐QTc interval, lower incidence of post B‐QTc prolongation, and higher incidence of postsevere B‐QTc prolongation as compared with subjects without severe B‐QTc lengthening. Meanwhile, severe F‐QTc lengthening had older age, longer post F‐QTc interval, lower incidence of post F‐QTc prolongation, and higher incidence of postsevere F‐QTc prolongation compared with subjects without severe F‐QTc lengthening.

**TABLE 5:** The comparison of characteristics based on severe B‐QTc lengthening and F‐QTc lengthening

Characteristics	Severe B‐QTc lengthening (n = 17)	No severe B‐QTc lengthening (n = 113)	*P* value	Severe F‐QTc lengthening (n = 16)	No severe F‐QTc lengthening (n = 114)	*P* value
Age (years), mean ± SD	53.1 ± 14.5	47.4 ± 15.9	.164^a^	56.8 ± 15.9	46.9 ± 15.5	.018
Male sex, n (%)	11 (64.7)	67 (59.3)	.671	11 (68.8)	67 (58.8)	.446
HCQ/CQ + AZT concomitant, n (%)	9 (52.9)	68 (60.2)	.571	10 (62.5)	67 (58.8)	.776
Treatments HCQ/CQ, n (%)			.308^b^			.090^b^
HCQ, n (%)	14 (82.4)	101 (89.4)		12 (75.0)	103 (90.4)	
CQ, n (%)	3 (17.6)	12 (10.6)		4 (25.0)	11 (9.6)	
HCQ/CQ duration (days), mean ± SD	5.6 ± 1.6	5.8 ± 1.5	.732^a^	5.8 ± 1.6	5.8 ± 1.4	.973^a^
HCQ/CQ cumdose (mg), mean ± SD	1888.2 ± 1261.9	1800.5 ± 1119.5	.766^a^	1956.3 ± 1290.9	1791.2 ± 1115.3	.588^a^
Tisdale score, mean ± SD	6.8 ± 2.2	6.9 ± 2.0	.814^a^	7.1 ± 2.6	6.9 ± 2.0	.707^a^
Tisdale risk category, n (%)			.896			.386
Low	9 (52.9)	53 (46.9)		9 (56.2)	53 (46.5)	
Moderate	7 (41.2)	52 (46.0)		5 (31.2)	54 (47.4)	
High	1 (5.9)	8 (7.1)		2 (12.5)	7 (6.1)	
COVID 19‐PCR positive, n (%)	9 (52.9)	80 (70.8)	.14	9 (56.2)	80 (70.2)	.262
Disease severity, n (%)						.584
Mild	4 (23.5)	46 (40.7)	.37	5 (31.2)	45 (39.5)	
Moderate	9 (52.9)	43 (38.1)		6 (37.5)	46 (40.4)	
Severe	4 (23.5)	24 (21.2)		5 (31.2)	23 (20.2)	
Hypertension (n, %)	4 (23.5)	33 (29.2)	.435	6 (37.5)	31 (27.2)	.392
Diabetes mellitus (n, %)	3 (17.6)	21 (18.6)	.615^b^	3 (18.8)	21 (18.4)	.601^b^
Ischemic heart disease, n (%)	1 (5.9)	3 (2.7)	.433^b^	0 (0)	4 (3.5)	.587^b^
Chronic heart failure (n, %)	1 (5.9)	5 (4.4)	.576^b^	1 (6.2)	5 (4.4)	.553^b^
Chronic kidney disease (n, %)	0 (0)	6 (5.3)	.424^b^	0 (0)	6 (5.3)	.447^b^
Creatinine, mean ± SD	1.8 ± 3.0	1.4 ± 1.9	.243^a^	2.3 ± 3.3	1.4 ± 1.8	.320^a^
Potassium, mean ± SD	4.2 ± 0.7	4.3 ± 0.6	.544	4.3 ± 0.7	4.3 ± 0.6	.959
Baseline heart rate (bpm), mean ± SD	96.59 ± 17.92	87.58 ± 17.32	.048	96.81 ± 17.49	87.62 ± 17.38	.05
Baseline B‐QTc (ms), mean ± SD	394.1 ± 57.5	425.4 ± 39.3	.005	NA	NA	NA
Baseline F‐QTc (ms), mean ± SD	NA	NA	NA	369.8 ± 56.0	400.9 ± 35.4	.045
Baseline B‐QTc prolongation, n (%)	2 (11.8)	25 (22.1)	.264^b^	NA	NA	NA
Baseline F‐QTc prolongation, n (%)	NA	NA	NA	2 (12.5)	6 (5.3)	.256^b^
Baseline severe B‐QTc prolongation, n (%)	1 (5.9)	5 (4.4)	.576^b^	NA	NA	NA
Baseline severe F‐QTc prolongation, n (%)	NA	NA	NA	0 (0)	0 (0)	NA
Post B‐QTc (ms), mean ± SD	489.6 ± 49.3	420.5 ± 39.9	<.001	NA	NA	NA
Post F‐QTc (ms), mean ± SD	NA	NA	NA	467.4 ± 60.1	401.4 ± 36.6	.001
Post B‐QTc prolongation, n (%)	12 (70.6)	21 (18.6)	<.001	NA	NA	NA
Post F‐QTc prolongation, n (%)	NA	NA	NA	7 (43.8)	9 (7.9)	<.001
Postsevere B‐QTc prolongation, n (%)	5 (29.4)	3 (2.7)	.001^b^	NA	NA	NA
Postsevere F‐QTc prolongation, n (%)	NA	NA	NA	4 (25.0)	1 (0.9)	.001^b^

Abbreviations: AZT, azithromycin; bpm, beat per minute; B‐QTc, corrected QT based on Bazett's formula; CQ, chloroquine; cum, cumulative; F‐QTc, corrected QT based on Fridericia's formula; HCQ, hydroxychloroquine; NA, not applicable; PCR, polymerase chain reaction; SD, standard deviation.

^a^ Mann–Whitney U‐test.

^b^ Fisher's Exact test.

The characteristics associated with severe B‐QTc and F‐QTc prolongation were depicted in Table 6. There was a significant shorter duration of HCQ/CQ treatment in severe F‐QTc prolongation compared with no severe F‐QTc prolongation. Subjects with severe B‐QTc and F‐QTc prolongations had longer QTc interval at baseline and posttreatment as well as higher incidence of QTc prolongation at baseline and posttreatment. Two subjects with severe QTc prolongation had experienced HCQ premature stop by their attending cardiologists, both were from the same hospital center. One subject was 25 year‐old male, who also had deteriorating atrioventricular block, and the other was 48 year‐old female, who also had hypokalemia. These cases had been reported elsewhere.^27^ No fatal arryhtmia occurred during this study.

**TABLE 6:** The comparison of characteristics based on severe B‐QTc prolongation and severe F‐QTc prolongation

Characteristics	Severe B‐QTc prolongation (n = 8)	No severe B‐QTc prolongation (n = 122)	*P* value	Severe F‐QTc prolongation (n = 5)	No severe F‐QTc prolongation (n = 125)	*P* value
Age (years), mean ± SD	51.3 ± 21.7	47.9 ± 15.4	.567	57.4 ± 22.9	47.8 ± 15.5	.182
Male sex, n (%)	6 (75.0)	72 (59.0)	.309^a^	3 (60.0)	75 (60.0)	.667
HCQ/CQ + AZT concomitant, n (%)	4 (50.0)	73 (59.8)	.422^a^	2 (40.0)	75 (60.0)	.32
Treatments HCQ/CQ, n (%)			.636^a^			.536^a^
HCQ, n (%)	7 (87.5)	108 (88.5)		5 (100.0)	110 (88.0)	
CQ, n (%)	1 (12.5)	14 (11.5)		0 (0.0)	15 (12.0)	
HCQ/CQ duration (days), mean ± SD	4.9 ± 1.4	5.8 ± 1.5	.065^b^	4.2 ± 1.3	5.8 ± 1.4	.003^b^
HCQ/CQ cumdose (mg), mean ± SD	1475.0 ± 747.9	1833.6 ± 1153.8	.219^b^	1520.0 ± 954.9	1823.2 ± 1142.5	.239^b^
Tisdale score, mean ± SD	7.9 ± 3.4	6.8 ± 2.0	.128^b^	8.6 ± 4.2	6.8 ± 1.9	.284^b^
Tisdale risk category, n (%)			.090^a^			.011^a^
Low	4 (50.0)	58 (47.5)		2 (40.0)	60 (48.0)	
Moderate	2 (25.0)	57 (46.7)		1 (20.0)	58 (46.4)	
High	2 (25.0)	7 (5.7)		2 (40.0)	7 (5.6)	
COVID 19‐PCR positive, n (%)	5 (62.5)	84 (68.9)	.489	3 (60.0)	86 (68.8)	.505^a^
Disease severity, n (%)			.257			.062
Mild	1 (12.5)	49 (40.2)		0 (0.0)	50 (40.0)	
Moderate	4 (50.0)	48 (39.3)		2 (40.0)	50 (40.0)	
Severe	3 (37.5)	25 (20.5)		3 (60.0)	25 (20.0)	
Hypertension (n, %)	3 (37.5)	34 (27.9)	.409^a^	2 (40.0)	35 (28.0)	.441^a^
Diabetes mellitus (n, %)	3 (37.5)	21 (17.2)	.164^a^	2 (40.0)	22 (17.6)	.229^a^
Ischemic heart disease, n (%)	0 (0)	4 (3.3)	.773^a^	0 (0.0)	4 (3.2)	.853^a^
Chronic heart failure (n, %)	1 (12.5)	5 (4.1)	.322^a^	1 (20.0)	5 (4.0)	.213^a^
Chronic kidney disease (n, %)	0 (0)	6 (4.9)	.678^a^	0 (0.0)	6 (4.8)	.787^a^
Creatinine, mean ± SD	3.0 ± 4.5	1.4 ± 1.8	.243^b^	3.8 ± 5.3	1.4 ± 1.8	.187^b^
Potassium,mean ± SD	4.2 ± 0.8	4.3 ± 0.6	.544	4.3 ± 1.0	4.3 ± 0.6	.978
Baseline heart rate (bpm), mean ± SD	94.25 ± 14.01	88.39 ± 17.79	.364	95.60 ± 14.43	88.48 ± 17.7	.377
Baseline B‐QTc (ms), mean ± SD	469.9 ± 45.8	418.1 ± 41.2	.001	NA	NA	NA
Baseline F‐QTc (ms), mean ± SD	NA	NA	NA	446.6 ± 48.7	395.2 ± 38.1	.004
Baseline B‐QTc prolongation, n (%)	5 (62.5)	22 (18.0)	.010^a^	NA	NA	NA
Baseline F‐QTc prolongation, n (%)	NA	NA	NA	3 (60.0)	5 (4.0)	.001^a^
Baseline severe B‐QTc prolongation, n (%)	3 (37.5)	3 (2.5)	.003^a^	NA	NA	NA
Baseline severe F‐QTc prolongation, n (%)	NA	NA	NA	0 (0)	0 (0)	NA
Post B‐QTc (ms), mean ± SD	541.8 ± 37.2	422.2 ± 37.6	<.001	NA	NA	NA
Post F‐QTc (ms), mean ± SD	NA	NA	NA	545.6 ± 47.5	404.1 ± 36.1	<.001
Post B‐QTc prolongation, n (%)	8 (100.0)	25 (20.5)	<.001^a^	NA	NA	NA
Post F‐QTc prolongation, n (%)	NA	NA	NA	5 (100.0)	11 (8.8)	<.001

Abbreviations: AZT, azithromycin; bpm, beat per minute; B‐QTc, corrected QT based on Bazett'sformula; CQ, chloroquine; cum, cumulative; F‐QTc, corrected QT based on Fridericia's formula; HCQ, hydroxychloroquine; NA, not applicable; PCR, polymerase chain reaction; SD, standard deviation.

^a^ Fisher's Exact test.

^b^ Mann–Whitney U‐test.

## 4 DISCUSSION

The results of our study corroborated several results of studies concerning HCQ/CQ treatment in COVID‐19. This study analyzed the QTc interval changes with two formulas: Bazett (B‐QTc) and Fridericia (F‐QTc). Both subjects with HCQ/CQ alone and HCQ/CQ + AZT concomitant treatment experienced QTc interval lengthening from baseline to posttreatment but significant QTc interval lengthening was detected in F‐QTc of subjects with HCQ/CQ + AZT concomitant treatment. The incidence of QTc prolongation, severe QTc lengthening, and QTc prolongation was not different in both HCQ/CQ alone and HCQ/CQ + AZT concomitant treatments.

Our study provided novel finding that Tp‐e interval was significantly and similarly increased after treatment in both HCQ/CQ alone and HCQ/CQ + AZT concomitant treatment. The Tp‐e/QT ratio only increased significantly in HCQ/CQ + AZT concomitant group. Our study did not find TdP or other life‐threatening and fatal arrhythmias. Therefore, overall, the treatment with HCQ/CQ‐contained regimens caused QTc interval changes and increased in Tp‐e interval and Tp‐e/QT ratio.

Previous prospective observational studies indicated similar results with our study, in which there was similar degree of QTc interval lengthening between COVID‐19 subjects receiving HCQ alone and HCQ + AZT combination treatment evaluated, and the incidence of QTc > 500 ms was 17.9%.^27^, ^28^ Another prospective study showed increasing in QTc interval in COVID‐19 subjects receiving HCQ + AZT, and its increases were temporally stable over the 24 hour.^29^, ^30^ This study showed very low incidence of QTc > 500 ms.^30^ One prospective study showed significant QTc lengthening and 2.8% incidence of QTc > 500 ms after 2 days HCQ + AZT treatment.^31^ By contrast, another study showed that HCQ/CQ + AZT had significantly longer final QTc interval and changes from final and baseline QTc interval as compared with HCQ/CQ alone.^25^ The number of patients with peak QTc > 500 ms were not significantly different.^25^ A prospective study confirmed that HCQ was harmless for COVID‐19 patients and did not associate with a risk of arrhythmia induced by QTc prolongation.^32^

Most data regarding evaluation of QTc interval prolongation after HCQ/CQ and AZT treatments come from China, South America, North America, and European countries. In our region, Southeast Asia, in which the prevalence of HCQ/CQ usage for malaria is frequent and despite its utilization in COVID‐19 national protocol, the prospective observational study resulting the data of HCQ/CQ safetyduring current COVID‐19 pandemic is sparse. There was only one study from one hospital in Malaysia, which was published and showed that confirmed COVID‐19 patients receiving HCQ with/without AZT had no significant QTc lengthening that peaked at day 4.^33^ The incidence of QTc prolongation was 38.5% without any TdP or other cardiac arrhytmias.^33^ The meta‐analysis which included studies until September 2020 concluded that HCQ/CQ‐associated cardiac toxicity in COVID‐19 was infrequent; however, it necessitated electrocardiogram monitoring especially in elderly and/or patients receiving other QT‐prolonging drugs, such as AZT.^34^, ^35^, ^36^, ^37^ A large, multicenter cohort study in Europe with various clinical settings, home management, medical ward, and ICU also revealed that HCQ in short‐term administration for COVID 19 only caused modest QTc increment and did not relate with arrhythmic death.^38^ Our study corroborated previous data and enriched the solid findings from the Southeast Asian region. Notably, subjects with concomitant AZT tend to be younger and have milder disease that those without AZT. As this was an observational study that was a coincidence, there was no tendency from physician to give concomitant AZT in younger and milder clinical presentation. However, there was a potential bias of data analysis due to these significant differences baseline data.

There is scarce evidence related to the effects of HCQ/CQ in Tp‐e interval changes in COVID‐19 patients. The underlying mechanism on how HCQ/CQ causes Tp‐e interval and Tp‐e/QT ratio increased, as observed in our study, has not been clearly elucidated.^39^ There are several hypothesizes underly why we choose Tp‐e and Tp‐e/QT ratio in this study, as stated by Prenneret al. (2016), the full repolarization of the M‐cell in the mid myocardial represents in the end of T wave; therefore, Tp‐e interval marks that the surface electrocardiogram corresponds to the dispersion of repolarization across the ventricular wall.^26^ The M‐cell itself has different electrophysiology properties compare with cells in the epicardium and endocardium, yet also has different response related to proarrhythmic drugs.^26^, ^40^ Consequently, in response to I_kr_ (potassium channel regulator) blockers, this M‐cell will have greater prolongation of action potensial duration then it will affect the overall myocardial repolarization.^26^, ^40^ Since HCQ/CQ is I_kr_ blocker, it is possible that the effect of quinidine toward Tp‐e interval is through this mechanism.^41^

The cut‐off points of Tp‐e interval or Tp‐e/QT ratio in relation to TdP are not yet established. Several studies propose different findings related to various condition.^22^, ^23^ Based on meta‐analysis of predictive value of Tp‐e indices in acquired QT prolongation pertaining to the incidence of TdP, the mean difference of Tp‐e interval and Tp‐e/QT ratio prolongation which related to TdP were 76 ± 26 ms and 0.14 ± 0.03, respectively.^42^ This study included drug‐induced QT prolongation.^42^ In our study, the mean differences of Tp‐e interval and Tp‐e/QT ratio were below the result of previous study which may explain why there was no incidence of TdP in our study. Furthermore, regimens which were used in this study, HCQ, CQ, and AZT, are classified in moderate risk for drug‐induced TdP.^43^

In this study, there was one arrhytmia observed, ie the deterioration of first‐degree AV block into second degree AV block during HCQ + AZT treatments. The worsening of AV block did not associate with prolonged QTc interval, but probably due to tachycardia induced by systemic infection or inflammation process. Both HCQ and CQ had effects on cardiac rhythm, conduction and duration of electrical activity.^42^, ^43^ This can be manifested as tachycardia, flattening of T wave, QTc prolongation and conduction block at level of sinus, atrioventricular (AV) node and ventricle.^41^ Besides mechanisms involving potassium channel and funny current, electrophysiologist side effects of HCQ/ CQ are related to daily cumulative dose and duration of treatment.^41^ The rare incidence of arrhythmia in our study was in line with findings of The Recovery Collaborative Group in which there were no significant differences of HCQ treatment and standard care on the low incidence of arrhythmia.^44^

### 4.1 Limitations of study

In this study, we could not examine objective data such as echocardiography and cardiac biomarkers to elucidate cardiac function of the patients and to exclude the concomitant myocarditis.

## 5 CONCLUSIONS

This prospective observational study indicates that HCQ/CQ + AZT concomitant treatment had significant posttreatment QTc interval changes toward F‐QTc lengthening. The HCQ/CQ alone and concomitant addition of AZT had similar incidences of severe QTc lengthening, QTc prolongation, and severe QTc prolongation. The incidences of severe QTc lengthening and severe QTc prolongation were low. A comparable significant increased in Tp‐e interval was observed in both HCQ/CQ alone and concomitant addition of AZT, from baseline to posttreatment. A Tp‐e/QT ratio was significantly increased only in HCQ/CQ + AZT concomitant treatment.

## CONFLICT OF INTEREST

Authors declare no conflict of interests for this article.
